# Study on the Performance of Composite-Modified Epoxy Resin Potting Adhesive for Repairing Oblique Cracks

**DOI:** 10.3390/ma18133197

**Published:** 2025-07-07

**Authors:** Zimin Chen, Zhengyi Li, Zhihong Ran, Yan Zhang, Fan Lin, Yu Zhou

**Affiliations:** School of Architecture and Planning, Yunnan University, Kunming 650500, China; m15205965692@163.com (Z.C.); ningan0302@163.com (Z.L.); overline999@163.com (Y.Z.); lin-fan@ynu.edu.cn (F.L.); 13173322663@163.com (Y.Z.)

**Keywords:** modified epoxy resin, grouting repair, diagonal crack, bending member

## Abstract

Reinforced concrete structures are prone to the development of microcracks during service. In this study, a composite-modified epoxy potting adhesive was formulated using nano-TiO_2_, carboxyl-terminated butadiene nitrile liquid rubber (CTBN), and the reactive diluent D-669. The mechanical properties and effectiveness of this composite adhesive in repairing oblique cracks were systematically evaluated and compared with those of single-component-modified epoxy adhesives. Key material parameters influencing the performance of oblique crack repair were identified, and the underlying repair mechanisms were analyzed. Based on these findings, a theoretical formula for calculating the shear-bearing capacity of beams with repaired web reinforcement was proposed. Experimental results demonstrated that compared to single-component-modified epoxy resin, the optimally formulated composite adhesive improved the tensile strength, elongation at break, and bond strength by 4.07–21.16 MPa, 13.28–20.4%, and 1.05–3.79 MPa, respectively, while reducing the viscosity by 48–872 mPa·s. The viscosity of the adhesive was found to play a critical role in determining the repair effectiveness, with toughness enhancing the crack resistance and bond strength contributing to the structural stiffness recovery. The adhesive effectively penetrated the steel–concrete interface, forming a continuous bonding layer that improved energy dissipation and significantly enhanced the load-bearing capacity of the repaired beams.

## 1. Introduction

Reinforced concrete is widely employed in the construction of bridges, tunnels, highways, and other infrastructure systems [1]. However, under the influence of sustained and excessive traffic loads, these structures are prone to developing microcracks (width < 1 mm), which can significantly compromise their structural integrity and long-term safety performance [2]. Currently, pressure grouting is the most commonly adopted technique for repairing such microcracks. The selection of an appropriate potting adhesive is critical, as it directly influences the restored structural load-bearing capacity and durability of the repaired component [3]. Although conventional epoxy resin potting adhesives exhibit favorable bonding strength and chemical stability [4,5,6], their high molecular weight and limited molecular chain flexibility result in suboptimal viscosity and toughness characteristics [7,8,9].

Current modification strategies for epoxy resin primarily involve the incorporation of additives such as nanoparticles [10], rubber particles [11], hyperbranched polymer particles [12], and reactive diluents [13,14]. However, each of these approaches presents inherent limitations, including insufficient toughening efficiency, increased viscosity, high production costs, and compromised mechanical strength. To overcome these challenges, the adoption of multi-component toughening strategies—aimed at achieving synergistic effects—has emerged as a prominent direction in epoxy resin modification research. For instance, Wang [15] reported that an epoxy resin modified with a CTBN to nano-CeO_2_ mass ratio of 4:1 exhibited a 692.27% increase in impact strength compared to the unmodified resin. Similarly, Wang [16] employed polysulfone (PSF) and nano-graphene oxide (GO) for composite modification, achieving an 89.90% enhancement in fracture toughness. Shi [17] demonstrated that the addition of two types of rubber additives not only improved the toughness of epoxy resin but also lowered its activation energy. Wang [18] further enhanced the thermal and mechanical properties of epoxy resin by incorporating liquid rubber and nano-alumina. Additionally, Wang [19] observed that the low- and room-temperature toughness of epoxy grout materials improved from 8.0% to 22.0% through the use of silicone and diluents. Although these studies confirm the potential of composite modification to enhance the mechanical performance of epoxy resins, the theoretical framework guiding such modifications remains underdeveloped. Moreover, limited attention has been given to the practical applications of modified epoxy resins in actual crack repair scenarios, thereby restricting their value and applicability in engineering practice.

Epoxy resin has been widely employed for crack repair in reinforced concrete structures, and numerous studies have reported favorable outcomes. For instance, Yin et al. [20] demonstrated that epoxy resin grouting significantly increased the initial cracking load of repaired reinforced concrete beams. Yuan et al. [21] conducted fatigue performance tests on main beams reinforced using epoxy resin grouting and found that this technique effectively restored structural stiffness and improved the bond strength at the steel–concrete interface. Similarly, BENGİ [22] investigated the flexural performance of cantilever beams repaired with epoxy resin under cyclic loading and observed that the resin facilitated a smoother transition between the elastic and inelastic regions of the load–deflection curve. However, these studies primarily focused on the repair of vertical cracks and did not address the repair of diagonal cracks. Furthermore, the fundamental mechanism by which epoxy infusion adhesives contribute to crack repair remains unclear, resulting in a relatively weak theoretical foundation in this area.

In response to the aforementioned limitations, carboxyl-terminated butadiene nitrile liquid rubber (CTBN) and nano-TiO_2_ were selected as toughening agents, while D-669 was employed as a reactive diluent to prepare a composite-modified epoxy resin potting adhesive. The formulation was optimized by evaluating the mechanical properties (tensile strength and elongation at break) and processing performance (bond strength and viscosity) of various compositions. To assess the practical effectiveness of the adhesive in structural applications, monotonic and cyclic loading tests were conducted to simulate crack development in reinforced concrete. The performance of the composite-modified adhesive was compared to that of single-component formulations with respect to their ability to repair diagonal cracks. Key material properties influencing repair performance were identified, and the underlying crack repair mechanisms of the modified epoxy resin potting adhesive were investigated. This study provides both theoretical insight and experimental evidence to support the composite modification of epoxy resin and offers a scientific basis for evaluating the repair effectiveness of grouting adhesives in the context of concrete diagonal crack restoration.

## 2. Materials and Methods

### 2.1. Raw Materials

E-51 is a kind of general bisphenol A liquid epoxy resin with the epoxy value of 0.51–0.54/100 g; nano-TiO_2_ is a toughening agent produced by Shanghai Meiyango Alloy Materials Co., Ltd. (Shanghai, China); CTBN is a toughening agent produced by Wengrui Chemical Guangzhou Co., Ltd. (Guangzhou, China); D-669 is ethylene glycol diglycidyl ether, which is used as a general reactive diluent and is able to reduce the viscosity of condensate of epoxy resin; 593 is an epoxy resin curing agent.

### 2.2. Preparation of Modified Epoxy Resin Potting Adhesive

**Figure 1:**
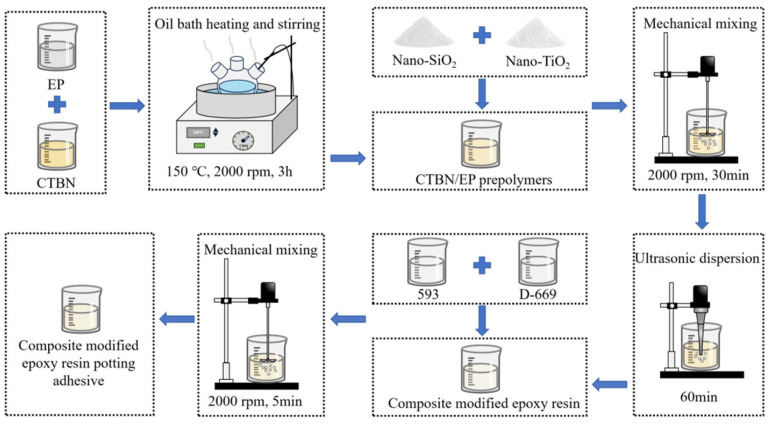
Manufacturing process for the modified epoxy resin potting adhesive.

Figure 1 presents the production process of the composite-modified epoxy resin potting adhesive. Initially, 100 g of epoxy resin E-51 was mixed with CTBN at mass fractions of 15%, 20%, and 25% relative to the epoxy resin in a three-necked flask. The mixture was heated in a silicone oil bath at 150 °C for 3 h to synthesize the CTBN/EP prepolymer. Subsequently, nano-TiO_2_ was added at mass fractions of 0.5%, 1.0%, and 1.5% relative to the epoxy resin. The mixture was mechanically stirred at 2000 rpm for 30 min, followed by ultrasonic dispersion for 60 min to ensure uniform distribution of nanoparticles and obtain the composite-modified epoxy resin. Finally, curing agent 593 (28 wt.% of the epoxy resin) and D-669 (6%, 9%, and 12 wt.% of the epoxy resin) were incorporated into the composite resin. The resulting mixture was mechanically stirred at 2000 rpm for 5 min to complete the preparation of the composite-modified epoxy resin potting adhesive.

### 2.3. Test Method

(1) Viscosity test

**Figure 2:**
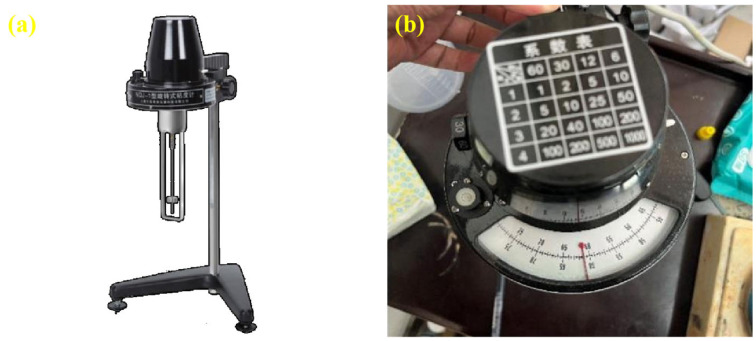
Viscosity test equipment. (**a**) viscosity test equipment; (**b**) viscosity test coefficient.

Viscosity is a critical indicator of the rheological behavior of potting adhesives. Lower viscosity corresponds to higher fluidity, which is essential for effective penetration and application [23]. In this study, the viscosity of the composite-modified epoxy resin potting adhesive at room temperature was measured using a rotary viscometer. The average viscosity over a 10 min interval was recorded and used as the evaluation criterion. The testing apparatus is shown in Figure 2.

(2) Bond performance test

**Figure 3:**
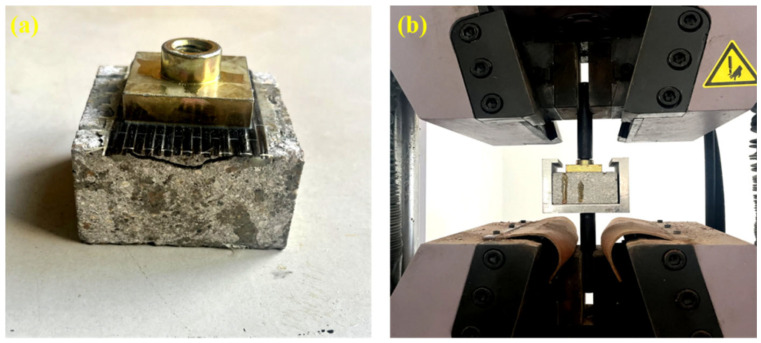
Testing process of bond performance: (**a**) specimens for bond performance tests; (**b**) bond performance test equipment.

Bond strength is a critical parameter for evaluating the adhesion performance of potting adhesives. Higher bond strength indicates a greater ability to resist secondary cracking [24]. In this study, carbon fiber cloth was embedded between a steel plate and a concrete block using the potting adhesive to simulate the bonding between reinforcement and concrete. The composite specimens were then placed in a testing mold, and the bond strength was measured under a loading rate of 3 mm/min. The dimensions of the steel plate and carbon fiber cloth were 40 mm in length and width, with the steel plate having a thickness of 10 mm. The concrete blocks measured 70 mm × 70 mm in surface area with a thickness of 40 mm. For each group, five specimens were tested. Outliers with significant deviations were excluded, and the average value of the remaining specimens was used as the final test result. The bond strength testing procedure is illustrated in Figure 3.

(3) Tensile properties test

**Figure 4:**
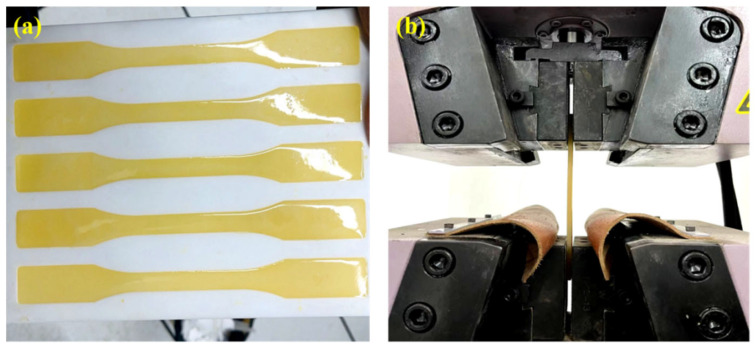
Testing process of tensile performance. (**a**) specimens for tensile property tests; (**b**) tensile property test equipment.

Tensile strength and elongation at break were used to evaluate the tensile properties of the potting adhesive [25]. The testing procedure followed the standard method specified in GB/T 2567-2021 for resin castings [26]. The adhesive was first injected into a standard mold, cured at ambient temperature (23 ± 2 °C) for 12 h, then demolded, and subjected to an additional curing period at the same temperature for 7 days. Tensile tests were conducted at a loading rate of 10 mm/min. The tensile specimens had a total length of 210 mm, a thickness of 4 mm, and a gauge length (marking distance) of 50 mm at the central section. For each formulation, five specimens were tested. Outliers with large deviations were excluded, and the average of the remaining results was recorded as the final test value. The testing procedure is illustrated in Figure 4.

(4) Oblique crack repair test

**Figure 5:**
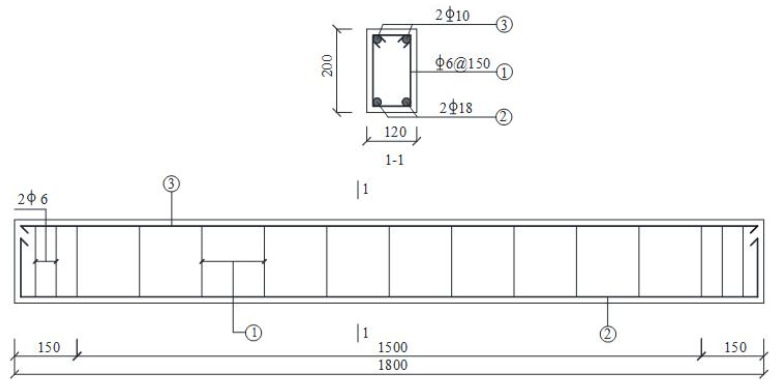
The dimensions and reinforcement of the reinforced concrete beam.

**Figure 6:**
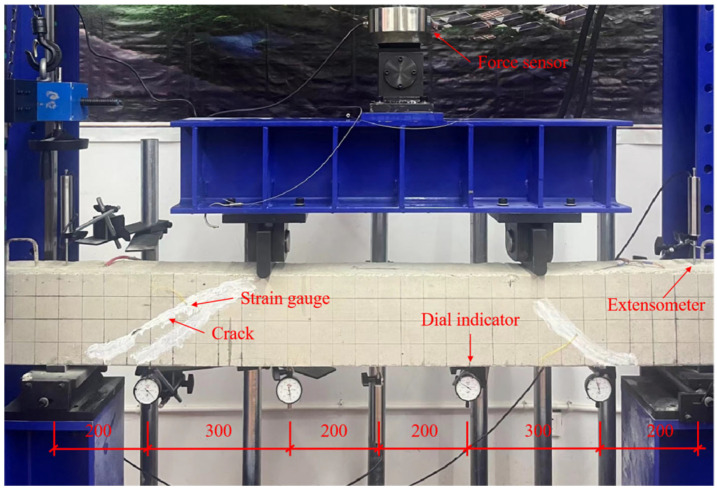
Illustration diagram of four-point bending loading of a specimen beam.

In this study, nine reinforced concrete beams were fabricated to evaluate the effectiveness of epoxy potting adhesives modified with different materials for repairing diagonal cracks. The dimensions and reinforcement details of the beams are shown in Figure 5. The longitudinal tensile reinforcement consisted of HRB400 hot-rolled ribbed bars with a yield strength of 400 MPa, while the hoop reinforcement used HPB300 hot-rolled plain bars, also with a yield strength of 400 MPa. The concrete used had a specified compressive strength grade of C25. Flexural loading was applied to the specimen beams using a four-point bending setup. During testing, various instruments were employed, including strain gauges, force sensors, dial indicators, extensometers, hydraulic jacks, and crack width gauges, to monitor structural responses. The arrangement of the testing instruments and loading apparatus is illustrated in Figure 6.

(5) SEM Analysis

To analyze the toughening mechanism of epoxy resin modified by different materials, the fracture surface of the specimen after tensile damage was plated with gold, and the fracture morphology of the specimen was tested by scanning electron microscopy (SEM). The equipment used was a German ZEISS Sigma300 (Brno, Czech Republic), with an accelerating voltage of 15.0 keV and an SEM magnification range of 2000×–33,333×.

## 3. Results and Discussion

### 3.1. The Effects of Different Materials on the Properties of Epoxy Resin

The effects of various modifiers on the tensile strength, elongation at break, bond strength, and viscosity of epoxy resin composites were systematically investigated through orthogonal experiments. The detailed experimental design is presented in Table 1.

(1) Effect of the preparation process on viscosity

**Figure 7:**
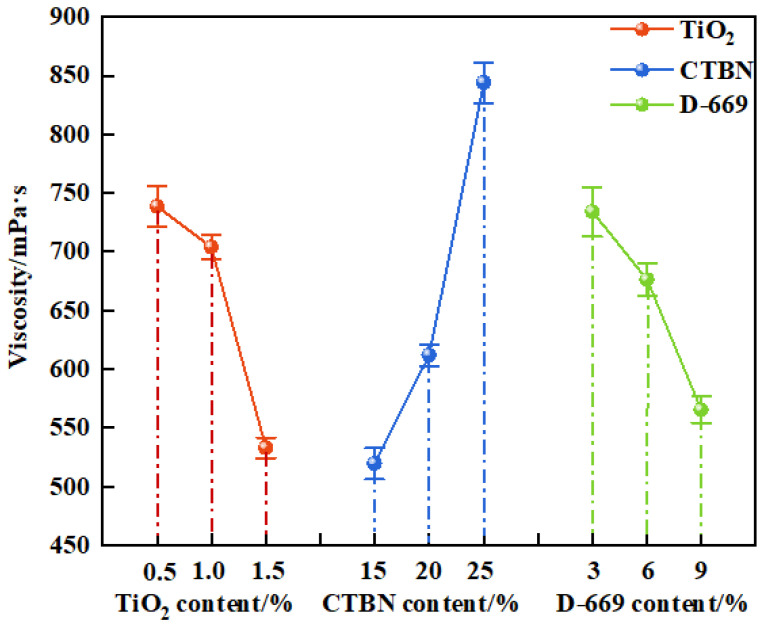
The viscosity of modified epoxy resin.

To investigate the effect of different preparation process parameters on the viscosity of the modified epoxy resin potting adhesive, the average viscosity over a 10 min interval was measured. The results are presented in Figure 7.

As shown in Figure 7, the viscosity of the modified epoxy resin potting adhesive decreases with increasing contents of nano-TiO_2_ and D-669 but increases with increasing CTBN content. The respective viscosity variation ranges caused by nano-TiO_2_, CTBN, and D-669 were 205.9 mPa·s, 324.7 mPa·s, and 168.7 mPa·s. This behavior can be attributed to the following mechanisms: The diluent D-669 reduces intermolecular interactions within the resin matrix, thereby lowering the overall viscosity; the inherently low surface energy of nano-TiO_2_ results in weaker particle–resin interactions, which also contributes to reduced viscosity. In contrast, CTBN, a high molecular weight polymer, introduces long-chain molecules that can entangle and partially crosslink with the epoxy resin chains, thereby enhancing intermolecular interactions and increasing the system’s viscosity [27].

(2) Effect of preparation process on bond performance

**Figure 8:**
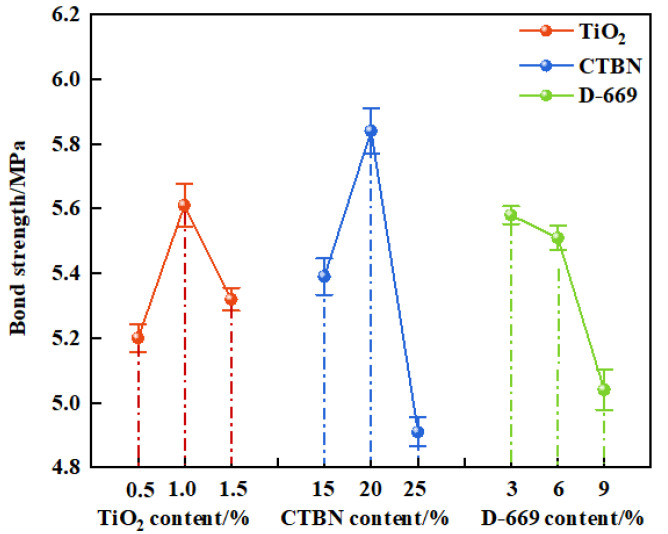
The bond performance of modified epoxy resin.

Bond strength was employed as the evaluation index to elucidate the influence of preparation process parameters on the bonding performance of the modified epoxy resin potting adhesive. The variation in bond strength under different formulation conditions was analyzed, as presented in Figure 8.

As shown in Figure 8, the bond strength of the modified epoxy resin potting adhesive initially increased and then decreased with increasing nano-TiO_2_ and CTBN contents, while it consistently decreased with increasing D-669 content. When the nano-TiO_2_ content reached 1%, the bond strength peaked at 5.61 MPa, representing increases of 0.41 MPa and 0.29 MPa compared to the formulations containing 0.5% and 1.5% nano-TiO_2_, respectively. This enhancement is attributed to the low surface energy of nano-TiO_2_ that improves the resin’s wettability and interfacial penetration, thereby enhancing adhesion. However, excessive nano-TiO_2_ content can lead to particle agglomeration on the cured resin surface, negatively impacting adhesion [11].

Similarly, the bond strength reached a maximum of 5.84 MPa at 20% CTBN content, increasing by 0.45 MPa and 0.93 MPa compared to those with 15% and 25% CTBN, respectively. This improvement is ascribed to the uniform dispersion of CTBN at lower contents, where significant energy is required to propagate cracks through the dispersed phase, thus enhancing bonding performance. In contrast, higher CTBN content results in the formation of a continuous rubbery phase, relegating the epoxy resin to the dispersed phase and thereby reducing bond strength.

In the case of D-669, the bond strength decreased to 5.04 MPa at a content of 12%, which is 0.54 MPa lower than that of the formulation with 6% D-669. This reduction indicates that excessive addition of the diluent compromises the cohesive strength of the epoxy network, thereby deteriorating the bonding performance of the potting adhesive.

(3) Effect of preparation process on tensile properties

**Figure 9:**
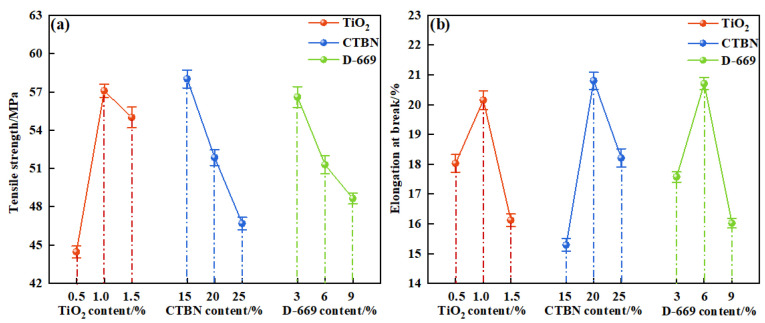
The tensile properties of modified epoxy resin: (**a**) The tensile strength of modified epoxy resin; (**b**) The elongation at break of modified epoxy resin.

The tensile properties of the modified epoxy resin potting adhesive were evaluated to investigate the effects of nano-TiO_2_ content, CTBN content, and D-669 content. The corresponding results are presented in Figure 9.

As shown in Figure 9, the tensile strength and elongation at break of the modified epoxy resin reached their maximum values when the nano-TiO_2_ content was 1%. Specifically, the tensile strength increased by 13.33 MPa and 2.09 MPa compared to those with 0.5% and 1.5% nano-TiO_2_ content, respectively. Similarly, the elongation at break improved by 2.12% and 4.03% under the same comparisons. This enhancement can be attributed to the reinforcing effect of rigid nanoparticles, which can improve the mechanical properties of epoxy resin. However, when the nanoparticle content exceeds a certain threshold, agglomeration tends to occur, which adversely affects the performance of the composite material [28,29].

When the CTBN content increased from 15% to 25%, the tensile strength of the modified epoxy resin decreased by 11.31 MPa. This reduction is attributed to the incorporation of CTBN, which disrupts the integrity of the epoxy resin’s cross-linked network [11], thereby reducing its strength. Notably, the elongation at break reached its maximum at a CTBN content of 20%, increasing by 5.51% and 2.59% compared to those of the resins containing 15% and 25% CTBN, respectively. This enhancement is primarily due to debonding and cavitation mechanisms occurring at lower CTBN contents, which absorb considerable energy and improve the toughness of the resin system [30]. However, when the CTBN content exceeds a certain threshold, the continuity of the epoxy matrix is compromised, ultimately leading to a reduction in elongation at break [31].

When the D-669 content increased from 6% to 12%, the tensile strength of the modified epoxy resin decreased by 7.94 MPa. This reduction is attributed to the presence of D-669, a linear small-molecule compound that diminishes the rigidity and strength of the epoxy resin network. At a D-669 content of 9%, the elongation at break reached its maximum, increasing by 3.13% and 4.68% compared to those of the resins with 6% and 12% D-669, respectively. These results indicate that an appropriate amount of D-669 can improve the flexibility of the epoxy resin, thereby enhancing its elongation at break.

### 3.2. Composition Optimization of Modified Epoxy Resin

As discussed in Section 3.1, excessive amounts of nano-TiO_2_, CTBN, and D-669 can adversely affect the properties of epoxy resin. To determine the optimal contents of individual and combined additives in the modified epoxy system, a gray relational analysis was performed. This method integrated the four performance indicators—tensile strength, elongation at break, bonding strength, and viscosity—into a single composite objective for comprehensive evaluation.

#### 3.2.1. Calculation of Gray Relational Degree

The procedure for computing the gray relational degree is as follows:

(1) Normalization: Multiple response values were normalized to eliminate the effect of dimensions on the analysis. The normalization formula is expressed in Equation (1).

(1) $$N=\frac{x-min(x)}{max(x)-min(x)}$$

where N is the normalized value of each response, max(x) is the maximum value of the actual response, min(x) is the minimum value of the actual response, and x is the actual value of each group of tests.

(2) Calculation of gray relational coefficient (GRC): GRC indicates the relationship between the experimental results and the optimal solution, and the calculation formulas are shown in Equations (2) and (3).

(2) $$GRC=\frac{\Delta_{min}+\zeta \Delta_{max}}{\Delta +\zeta \Delta_{max}}$$

(3) Δ=1−N

where ∆ represents the deviation sequence; ζ is the judgment coefficient, ζ ∈ [0, 1]; and in this study, ζ is 0.5.

(3) Response weight calculation: In order to balance the strength and toughness of the multi-additive-modified epoxy resin, the weights of each response were set to be the same.

(4) Calculation of gray relational degree (GRG): GRG is the weighted sum of gray relational coefficients; the formula for GRG (see Equation (4)) is given as follows:

(4) $$GRC=\sum_{i=1}^{n}\beta_{i}GRC$$

where β_i_ is the weight of the response. In this study, the weight ratios between tensile strength, elongation at break, bond strength, and viscosity were set to 1:1:1:1, resulting in β_i_ = 0.25.

#### 3.2.2. Determination of the Optimal Content of Composite-Modified Epoxy Resin

Based on the data presented in Table 1, the gray relational degrees of modified epoxy resin formulations with varying additive ratios were calculated, and the results are summarized in Table 2. A higher gray relational degree corresponds to superior overall material performance. As shown in Table 2, the maximum gray relational degree was achieved when the contents of nano-TiO_2_, CTBN, and D-669 were 1%, 20%, and 9%, respectively, indicating that this combination yields the optimal comprehensive performance.

#### 3.2.3. Determination of the Optimal Content of Single Additive-Modified Epoxy Resin

The tensile strength, elongation at break, bonding strength, and viscosity of epoxy resins modified with varying additive contents were evaluated. The gray relational degree was employed as a comprehensive evaluation index to assess the overall performance of the modified epoxy resin and to determine the optimal content of each additive. The detailed experimental design and the corresponding gray relational analysis results are presented in Table 3.

As shown in Table 3, the gray relational degree of the nano-TiO_2_-modified epoxy resin reaches its maximum at a nano-TiO_2_ content of 1%. Similarly, the gray relational degree of the CTBN-modified epoxy resin is highest at a CTBN content of 20%, while that of the D-669-modified epoxy resin peaks at a D-669 content of 9%. Therefore, the optimal comprehensive performance of the modified epoxy resin potting adhesive is achieved when the contents of nano-TiO_2_, CTBN, and D-669 are 1%, 20%, and 9%, respectively.

### 3.3. Comparison of the Properties of Modified Epoxy Resins

**Figure 10:**
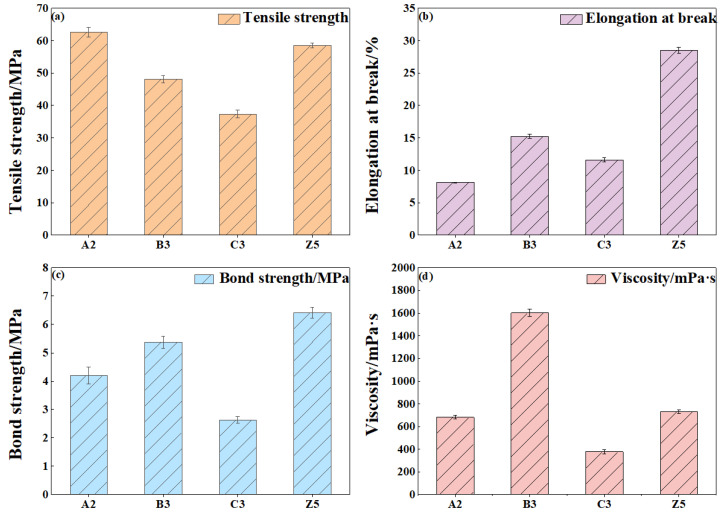
Comparison of the properties of epoxy resins modified by different materials: (**a**) tensile strength; (**b**) elongation at break; (**c**) bond strength; and (**d**) viscosity.

According to Section 3.2.2 and Section 3.2.3, the modified epoxy resin exhibits optimal comprehensive performance when the contents of TiO_2_, CTBN, and D-669 are 1%, 20%, and 9%, respectively. To evaluate the performance differences between single-additive and multi-additive-modified epoxy resins, the mechanical and rheological properties (including tensile strength, elongation at break, bond strength, and viscosity) were compared, as illustrated in Figure 10.

As shown in Figure 10, specimen A2 exhibits the highest tensile strength, reaching 62.6 MPa. The tensile strength of specimen Z5 is 4.07 MPa lower than that of A2. This difference is attributed to the partial suppression by nano-TiO_2_ of the strength-reducing effects caused by CTBN and D-669, resulting in the tensile strength of the composite-modified epoxy resin being slightly lower than that of the nano-TiO_2_-modified resin.

Z5 also demonstrates the highest elongation at break among the four formulations, reaching 28.52%. This is due to the fact that the elongation at break of all three individual components (nano-TiO_2_, CTBN, and D-669) peaks at these respective contents. In addition, synergistic effects among the three additives may further contribute to the enhanced toughness of the composite-modified epoxy resin.

The reinforcing effects of nano-TiO_2_ and CTBN on the bonding strength are greater than the weakening influence of D-669, enabling the composite formulation to maintain excellent adhesive strength. Furthermore, the viscosity of Z5 is reduced by 872 mPa·s compared to that of B3, indicating that the combined use of nano-TiO_2_, CTBN, and D-669 effectively mitigates the excessive viscosity typically introduced by CTBN alone.

In summary, a comprehensive analysis of tensile strength, elongation at break, bonding strength, and viscosity indicates that the composite-modified epoxy resin outperforms those modified with single additives, offering superior overall performance.

### 3.4. Comparison of Repair Effects on Diagonal Cracks

To evaluate the effectiveness of modified epoxy resin grouting adhesives containing different materials for repairing diagonal cracks, nine reinforced concrete beams were fabricated for experimental testing. Beam B-1 served as the control specimen and did not undergo any grouting repair. For beams B-2, B-3, B-4, and B-5, loading was applied at a constant force of 2 kN until diagonal cracks with widths ranging from 0.2 mm to 0.5 mm developed. These beams were subsequently repaired using different grouting materials. After the adhesives were fully cured, all specimens were reloaded following the same procedure used for B-1. The detailed repair scheme is presented in Table 4.

#### 3.4.1. Crack Development

**Figure 11:**
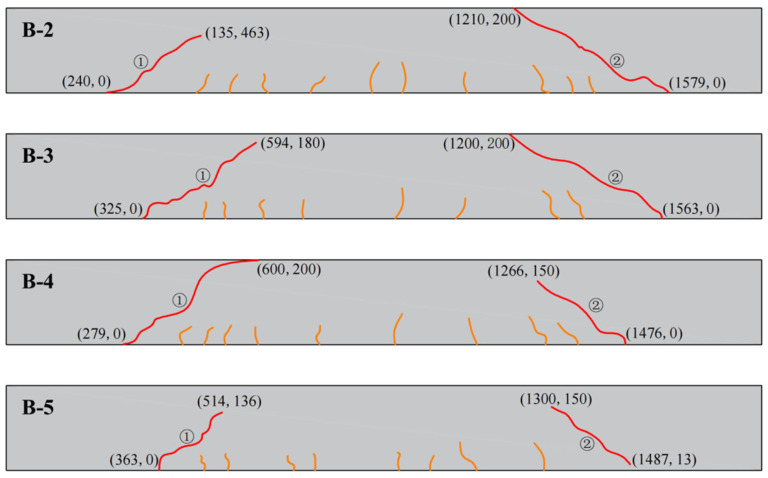
Crack development before repair.

**Figure 12:**
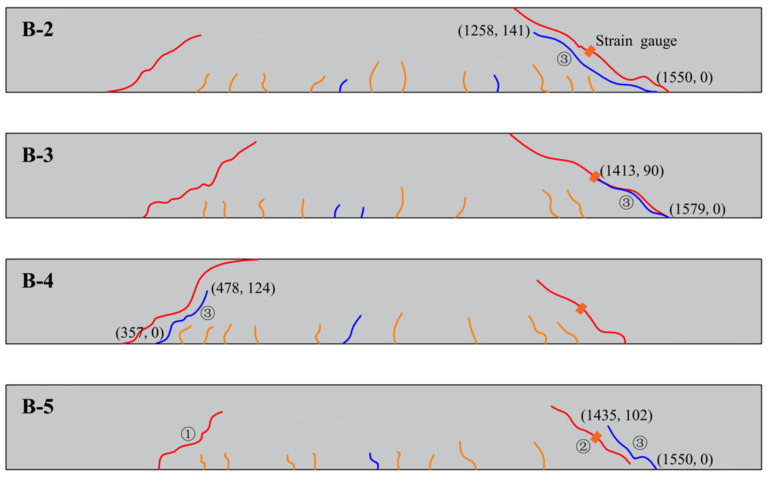
Crack development after repair.

The development of cracks before and after the repair of experimental beams using modified epoxy resin potting adhesives with different materials is illustrated in Figure 11 and Figure 12. During the early stages of loading, flexural cracks gradually appeared at the bottom of the beam specimens. As the load increased, the number of flexural cracks stabilized, while diagonal cracks began to form in the shear span. Multiple diagonal cracks intersected and extended, ultimately leading to the formation of dominant diagonal cracks.

The coordinate system is defined with the origin located at the lower-left corner of the beam. A Cartesian coordinate system is established, with the X-axis directed horizontally to the right and the Y-axis oriented vertically upward along the beam. The starting and ending points of cracks correspond to the recorded coordinates of crack initiation and termination. Cracks labeled as ① and ② were generated during the initial loading and subsequently filled with adhesive. Crack ③ was induced during secondary loading. The remaining cracks were annotated to indicate closure after repair.

As shown in Figure 11 and Figure 12, no new crack propagation was observed at the original oblique crack locations during secondary loading after the reinforced concrete beams were repaired using nano-TiO_2_-modified epoxy resin, D-669-modified epoxy resin, or the composite-modified epoxy resin. In contrast, for beams repaired with CTBN-modified epoxy resin, the cracks that formed during secondary loading overlapped with the original crack paths.

This difference may be attributed to the high permeability of low-viscosity epoxy resin adhesives, which not only fill the cracks effectively but also penetrate into the adjacent concrete under pressure, thereby enhancing the crack resistance of the surrounding region. In comparison, CTBN, due to its high molecular weight, significantly increases the viscosity of the modified epoxy resin, limiting its ability to infiltrate the cracks through pressure grouting and thus reducing the effectiveness of oblique crack repair.

#### 3.4.2. Expansion of Cracks During Secondary Loading

**Figure 13:**
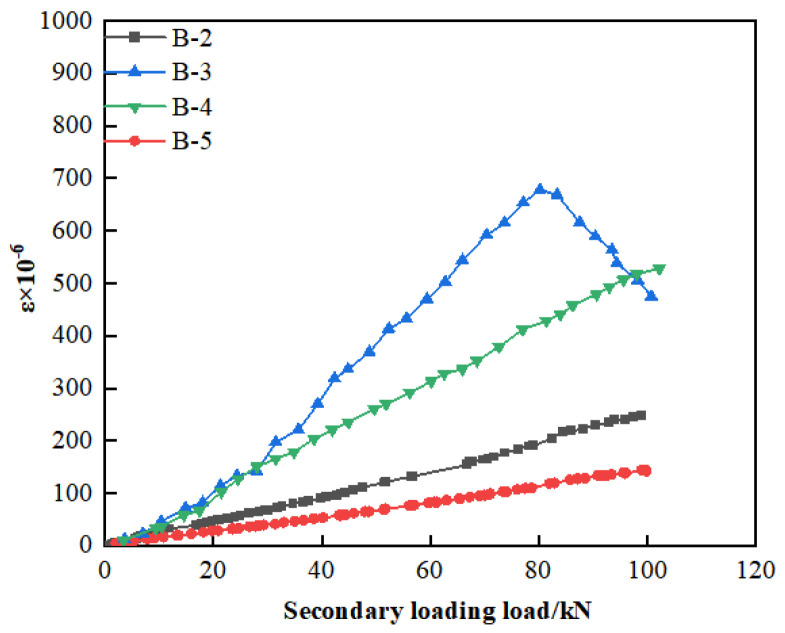
The load–strain curves of cracks.

To evaluate the effectiveness of modified epoxy resin potting adhesives formulated with different materials for crack repair, this study investigated crack propagation behavior under secondary loading by attaching strain gauges on both sides of the cracks. The sum of the measured strains was used as a parameter to characterize crack extension. The arrangement of the strain gauges is shown in Figure 12, and the corresponding load–strain curves for each crack are presented in Figure 13.

As illustrated in Figure 13, the strain in beam B-3 increases continuously with the applied load until it reaches 82 kN. At this point, a turning point appears in the load–strain curve, after which the strain begins to decrease despite further increases in load. The emergence of this turning point suggests the initiation of secondary cracking at the original crack locations [32]. This behavior may be attributed to the limited crack-repair capability of B-3, whereby the potting adhesive fails to effectively arrest crack propagation under increasing load, ultimately resulting in re-cracking of the repaired region.

The crack strain in beams B-2, B-4, and B-5 increases continuously with the applied load, without exhibiting any downward trend. This suggests that after crack repair using A2, C3, and Z5, the original cracks continued to open under secondary loading but did not undergo failure. This behavior may be attributed to the ability of nano-TiO_2_, CTBN, and D-669 to effectively enhance the toughness of the adhesive. The elongation at break of A2, C3, and Z5 was 8.12%, 11.63%, and 28.52%, respectively, all significantly exceeding the minimum requirement of 1.7% specified in GB/T 2567-2021 [24]. This improved ductility contributes to better deformation compatibility in the repaired concrete sections and reduces the risk of brittle failure. As a result, no secondary cracking was observed in the beams repaired with these three types of adhesives.

Furthermore, the average crack strain of beam B-5 was reduced by 29.55 and 206.34 microstrain compared to beams B-2 and B-4, respectively. This reduction may be attributed to the excellent fluidity of Z5, which enables it to effectively penetrate and fill the cracks. In addition, Z5 integrates the high tensile strength of nano-TiO_2_ with the superior bonding capacity of CTBN, thereby providing enhanced resistance to crack propagation along the direction of principal tensile stress.

#### 3.4.3. Initial Cracking Load and Ultimate Load

**Figure 14:**
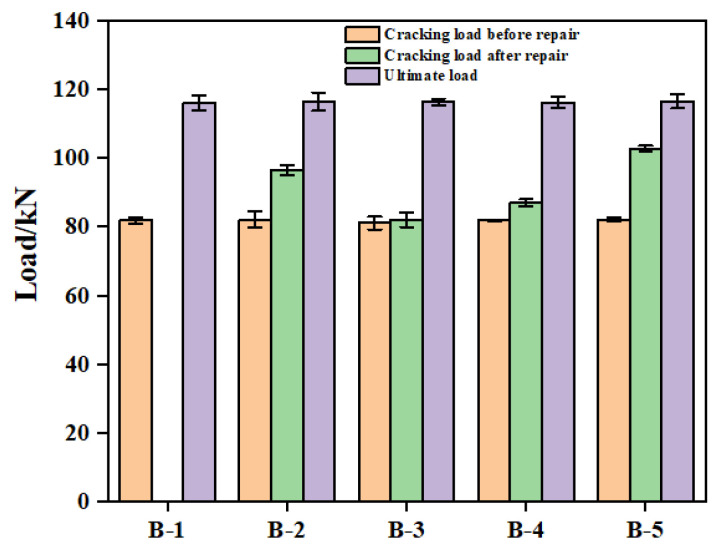
Cracking loads of the bending-shear sections and ultimate loads of the beams.

Figure 14 presents the cracking and ultimate loads of the bending-shear sections for various reinforced concrete beams. As shown in the figure, the initial cracking loads of all five beams were approximately equal, each around 82 kN. Following repair with epoxy resins modified using different materials, the cracking loads during secondary loading for beams B-2, B-3, B-4, and B-5 increased to 96.54 kN, 82.05 kN, 89.92 kN, and 102.73 kN, respectively. These values correspond to increases of 14.48 kN, 1.02 kN, 5.11 kN, and 20.71 kN compared to their respective pre-repair levels. These results indicate that the application of potting adhesives A2, C3, and Z5 effectively enhanced the crack resistance of the repaired bending-shear sections. In contrast, the adhesive B3 exhibited negligible improvement in crack resistance. This outcome is consistent with the observation of secondary cracking in beam B-3 and is attributed to the high viscosity of CTBN. The elevated viscosity of B3 likely inhibited its penetration into the surrounding concrete matrix, thereby diminishing its effectiveness in reinforcing the cracked region compared to the other adhesives.

The ultimate cracking loads for beams B-1, B-2, B-3, B-4, and B-5 were 116.17 kN, 116.42 kN, 116.37 kN, 116.21 kN, and 116.56 kN, respectively, demonstrating a high degree of consistency. This result is consistent with that observed by Yin [20], suggesting that the modified epoxy resin potting adhesives primarily enhance the structural performance within the load range between the initial cracking load and the ultimate load. In contrast, the ultimate load-bearing capacity of the reinforced concrete beams is predominantly governed by their internal reinforcement configuration rather than the adhesive properties.

#### 3.4.4. Beam Displacement

**Figure 15:**
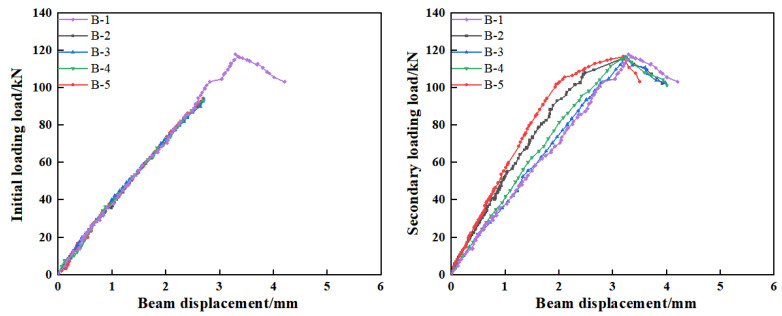
The positional relationship between the secondary loading cracks and the primary loading cracks.

Figure 15 illustrates the load–midspan deflection curves of the beams during both the initial and secondary loading processes. As shown, the deflection responses of all five beams were nearly identical during the initial loading stage. During secondary loading, the average midspan deflections of beams B-1, B-2, B-3, B-4, and B-5 were 1.67 mm, 1.13 mm, 1.66 mm, 1.57 mm, and 1.00 mm, respectively. Compared to the unrepaired state, the midspan deflection of beam B-3 decreased by only 0.13 mm. This limited improvement suggests that an excessively high viscosity of the potting adhesive may impede its penetration and bonding effectiveness, thereby restricting the restoration of the beam’s stiffness.

The average midspan displacement of beam B-5 was 0.13 mm lower than that of beam B-2. As discussed in Section 3.3, both A2 and Z5 exhibit comparable fluidity and possess excellent tensile strength. The elongation at break of the adhesive is primarily associated with the occurrence of secondary cracking within the structure. Therefore, it can be inferred that the bonding strength of the adhesive plays a critical role in effectively repairing beam damage and enhancing the structural load-bearing capacity. In contrast, the average midspan displacement of beam B-4 increased by 0.57 mm compared to that of beam B-5. This can be attributed to the fact that although C3 exhibits better fluidity than A2 and Z5, its relatively low tensile and adhesive strengths result in inadequate reinforcement in the cracked regions of the concrete.

### 3.5. Repair Effects Against Cyclic Loading

**Figure 16:**
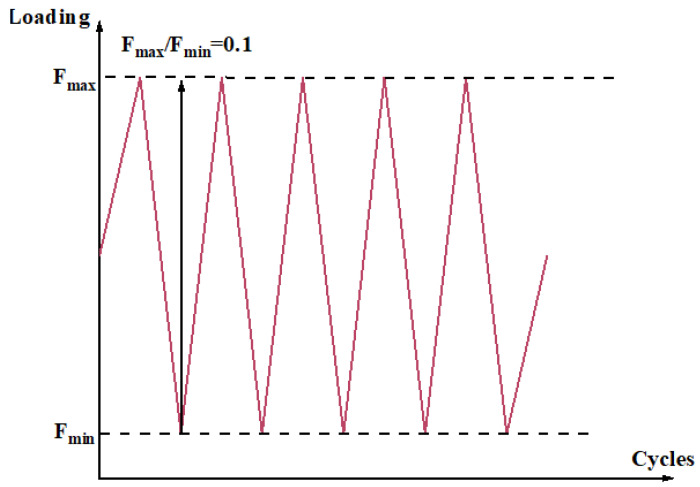
Loading method for low-cycle fatigue tests.

The repeated application of vehicular loads, coupled with temperature fluctuations, renders bridge structures vulnerable to fatigue damage over prolonged service periods, thereby raising safety concerns. To assess the effectiveness of epoxy resin adhesives modified with various materials in repairing beams subjected to low-cycle fatigue damage, low-cycle fatigue loading tests were performed on beams B-6, B-7, B-8, and B-9. The evaluation was based on changes in beam stiffness before and after repair. Using the shear-bearing capacity (F_0_) of the test beams as a reference, fatigue loading was applied in descending levels, with F_max_/F_0_ values of 0.8, 0.7, 0.6, and 0.5. The minimum load F_max_/F_0_ was set at 10% of the corresponding maximum load F_max_/F_0_, maintaining an F_max_/F_0_ ratio of 0.1. The loading protocol is illustrated in Figure 16.

**Figure 17:**
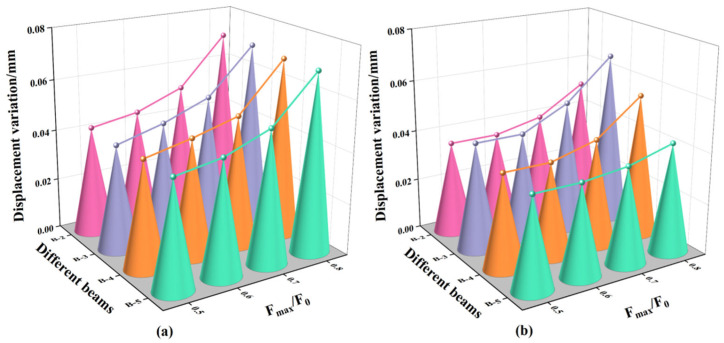
Linear relationship between the loading cycles and the mid-span displacement: (**a**) The displacement variation of the beam specimen before repair; (**b**) The displacement variation of the beam specimen after repair.

The mid-span section was selected as the control section, and the corresponding displacement variations of the beams before and after repair are presented in Figure 17. As the ratio of F_max_/F_0_ increased from 0.5 to 0.8, the displacement variation prior to repair increased from 0.028 mm to 0.030 mm. This trend indicates that higher fatigue loading levels accelerate the accumulation of fatigue-induced damage, resulting in greater displacement variations in the beam structure.

Following repair with different adhesives, the displacement variations of beams B-2, B-4, and B-5 exhibited no significant reduction at F_max_/F_0_ = 0.5 and 0.6, a slight reduction at F_max_/F_0_ = 0.7, and a substantial reduction at F_max_/F_0_ = 0.8, with decreases of 38.6%, 20.0%, and 28.2%, respectively, compared to their pre-repair states. This phenomenon may be attributed to the limited energy release within cracks under lower fatigue loads, which is insufficient to fully activate the adhesive’s bonding potential. As the fatigue load increases, significant stress concentrations—particularly at crack tips—enhance crack activation. Under such conditions, low-viscosity adhesives can effectively penetrate the cracks, improve bonding performance, strengthen the crack interface, reinforce the repaired concrete region, and ultimately enhance the structure’s fatigue resistance.

In addition, the displacement variation of beam B-5 was 15.6% lower than that of beam B-2. This observation aligns with the trend observed under monotonic loading, where beam B-5 exhibited smaller displacement than beam B-2. The discrepancy is attributed to the differences in bonding strength between adhesives Z5 and A2, which result in varying levels of stiffness recovery. However, due to the limited number of fatigue cycles, the observed differences in displacement under fatigue loading were less pronounced than those under monotonic loading conditions.

For beam B-3, the displacement variations decreased by 0.000 mm, 0.004 mm, 0.002 mm, and 0.004 mm at F_max_/F_0_ levels of 0.5, 0.6, 0.7, and 0.8, respectively. These minimal reductions are attributed to the high viscosity of adhesive Z5, which hindered its ability to adequately penetrate and repair the internal cracks, thereby limiting its effectiveness in restoring structural stiffness under fatigue loading.

### 3.6. Mechanism of Repairing Oblique Cracks

#### 3.6.1. Toughening Mechanism Analysis

**Figure 18:**
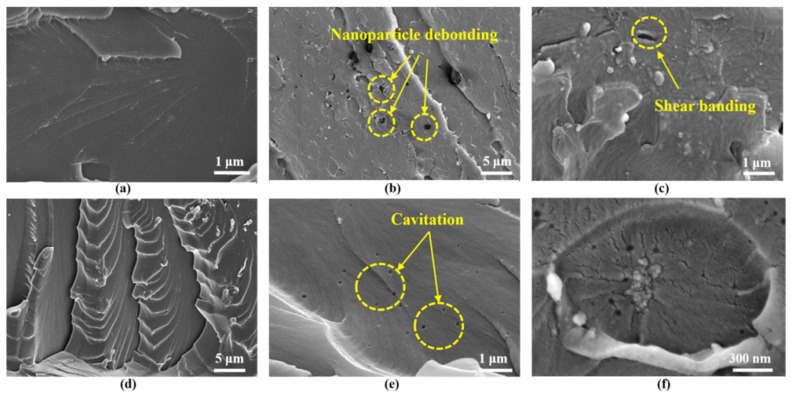
Scanning electron microscope test result: (**a**) Pure epoxy resin; (**b**) and (**c**) A2; (**d**) C3; (**e**) B3; (**f**) Z5.

As discussed above, nano-TiO_2_, CTBN, and D-669 can act as effective toughening agents for epoxy resin, enhancing the elongation at break of the modified epoxy potting adhesives. This improvement contributes to the prevention of secondary cracking in the repaired beams. To further investigate the toughening mechanisms of these three agents, scanning electron microscopy (SEM) analysis was performed on the fracture surfaces of tensile specimens made from pure epoxy resin, as well as specimens A2, B3, C3, and Z5 after tensile failure. The SEM results are presented in Figure 18.

Figure 18a shows the impact fracture surface of pure epoxy resin, which appears notably smooth with a uniform crack propagation direction, indicating a typical brittle fracture mode. In contrast, Figure 18b,c reveal that the fracture surfaces of epoxy resin modified with nano-TiO_2_ are significantly rougher and contain numerous voids, resulting from shear band formation and nanoparticle debonding; this phenomenon is consistent with Zewde’s observations [33]. This increased surface roughness promotes the formation of plastic cavities within the resin matrix and enhances the material’s ability to absorb deformation energy, thereby contributing to improved toughness.

The incorporation of D-669 induces partial phase separation from the epoxy matrix, resulting in the formation of micro-phase boundaries. As a crack propagates and encounters these interfaces, it deviates from its original linear path, as illustrated in Figure 18d, thereby consuming additional energy and effectively retarding crack growth. Figure 18e displays the fracture surface of epoxy resin modified with CTBN, characterized by numerous uniformly distributed spherical voids throughout the resin matrix. These voids, formed through cavitation initiated by the CTBN particles, function as stress dispersion and energy absorption sites, thereby impeding crack propagation and enhancing the overall toughness of the material [34,35].

The study by Wang et al. [15] demonstrates that nanoparticles and CTBN can synergistically enhance the toughness of epoxy resins. Specifically, nano-TiO_2_ particles can adsorb onto the surfaces of CTBN domains, facilitating localized plastic deformation around these regions and leading to the formation of cavities, as illustrated in Figure 18f. These cavities act as additional toughening mechanisms by blunting crack tips and increasing the crack propagation path, thereby improving the overall fracture resistance of the epoxy matrix.

#### 3.6.2. Permeability Analysis

**Figure 19:**
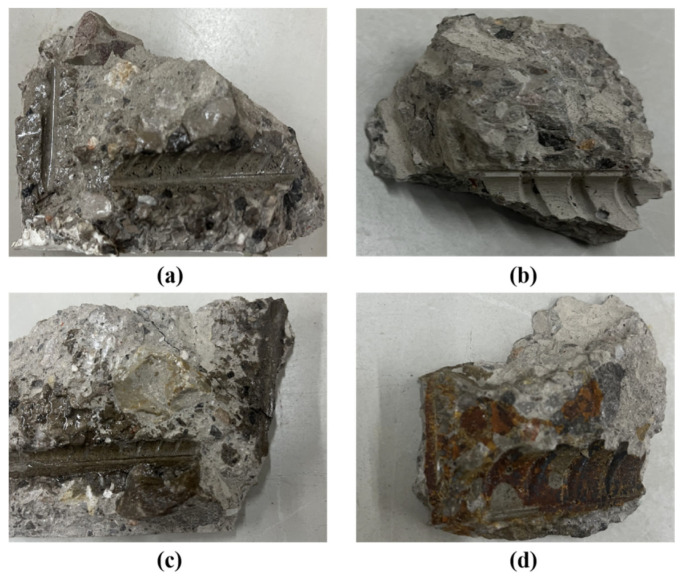
Adhesive penetration: (**a**) A2; (**b**) B3; (**c**) C3; (**d**) Z5.

To examine the penetration behavior of different infusion adhesives within cracks, concrete specimens were extracted from the intersection of stirrups and longitudinal reinforcement at Crack 1 of beams B-2, B-3, B-4, and B-5. The sampling results are presented in Figure 19.

As shown in Figure 19, the potting adhesives A2, C3, and Z5 successfully penetrated the interior of the cracks following pressure grouting and continued to spread along the crack propagation path, reaching the intersection of the cracks with the stirrups and longitudinal reinforcement. The CTBN component in Z5 contains unsaturated bonds that may undergo mild crosslinking reactions over time, potentially leading to a darkening of the adhesive color—a phenomenon considered normal and not indicative of performance degradation. In contrast, adhesive B3 exhibited poor penetration into the cracks, which is attributed to its high viscosity. This observation corroborates the previously discussed hypothesis regarding the inferior crack resistance and structural stiffness recovery observed in beam B-3. These findings underscore the critical importance of adhesive fluidity in ensuring effective crack repair. Without sufficient fluidity, even adhesives with favorable mechanical properties may fail to perform effectively in practical applications.

#### 3.6.3. Mechanism of Adhesion

**Figure 20:**
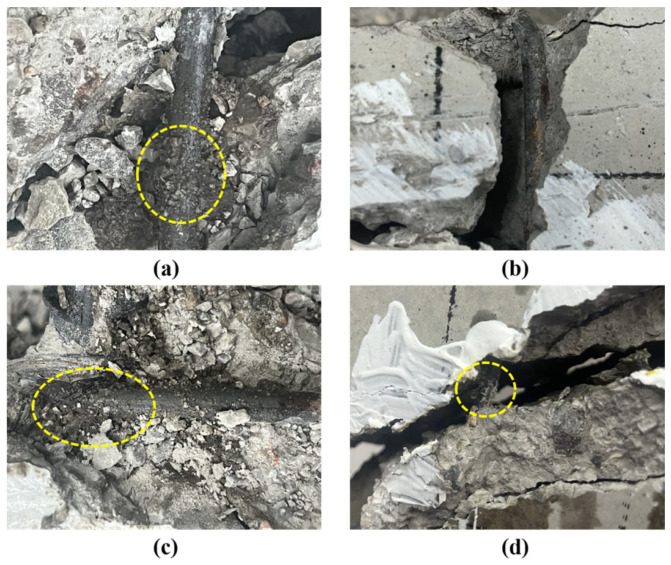
Adhesive penetration at the hoop: (**a**) A2; (**b**) B3; (**c**) C3; (**d**) Z5.

As previously discussed, potting adhesives can effectively repair beam damage and inhibit secondary cracking in structural elements. To further investigate the bonding performance of the adhesives at crack interfaces, beams B-2, B-3, B-4, and B-5 were sectioned along Crack ①, as illustrated in Figure 20.

As shown in Figure 20, the hoop interface in beam B-3 appears relatively smooth, whereas a distinct bonding layer is observed at the hoop interfaces of beams B-2, B-4, and B-5. This bonding layer is formed by a combination of concrete debris (generated during loading as a result of relative movement between the hoops and the surrounding concrete) and adhesive that infiltrated the hoop surface. Previous studies have shown that the bonding layer may contribute to chemical bonding at the reinforcement–concrete interface, thereby restoring the integrity of the cracked concrete and enabling it to collaborate with the hoops in resisting shear forces [21], but this conclusion has not been proven.

**Figure 21:**
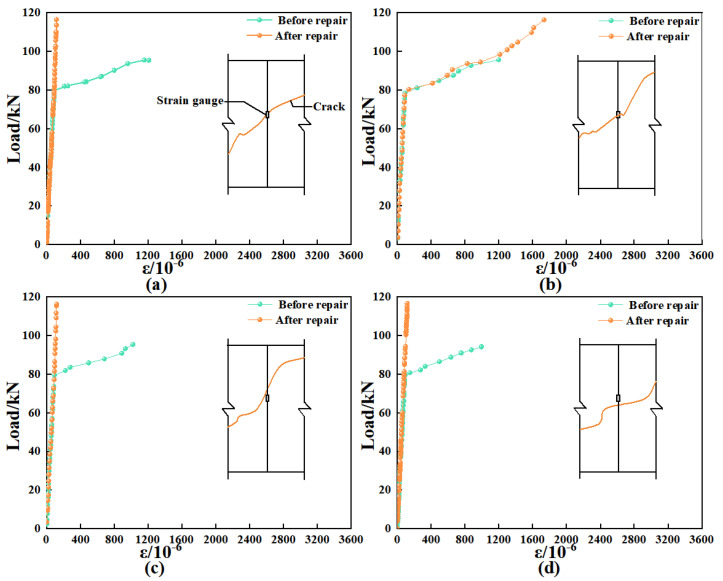
Hoop strain: (**a**) A2; (**b**) B3; (**c**) C3; (**d**) Z5.

To verify the function of the bonding layer, the hoop strain in beams B-2, B-3, B-4, and B-5 was analyzed under both the initial and secondary loading stages after repair, as shown in Figure 21. During the initial loading phase, most of the shear force was carried by the concrete, resulting in minimal changes in the hoop strain across all four beams. As loading progressed and the beams reached their respective cracking loads, hoop strain increased sharply with the development of diagonal cracks, eventually leading to the formation of critical diagonal cracks. At this stage, the peak stirrup strains recorded during the first loading phase were 1210.73, 1198.40, 1021.56, and 996.46 for beams B-2, B-3, B-4, and B-5, respectively. These results indicate that hoop strain is more significantly influenced by cracking when the stirrup is located closer to the critical diagonal crack.

Following repair with potting adhesives A2, B3, C3, and Z5, the peak hoop strains during the secondary loading phase were 120.32, 1417.82, 119.53, and 122.16 for beams B-2, B-3, B-4, and B-5, respectively. As shown, beam B-3 exhibited a significantly higher hoop strain during secondary loading, which can be attributed to the reopening of the original crack. In contrast, beams B-2, B-4, and B-5 demonstrated substantially lower hoop strains, indicating effective crack repair. In these beams, the adhesives permeated the interface between the hoop and the surrounding cracked concrete, forming a bonding layer.

This phenomenon shows that the bonded layer can bear most of the shear force together with the repaired concrete during the secondary loading process, thus reducing the load transferred to the hoop reinforcement and limiting the increase in hoop strain, which verifies the hypothesis about the role of the bonded layer proposed by Yuan [21].

Throughout both loading phases, the stirrups remained within the elastic range and experienced only elastic deformation without yielding. This observation provides a theoretical foundation for deriving a shear capacity calculation formula for web-reinforced beams repaired using adhesive grouting.

#### 3.6.4. Shear-Bearing Mechanism

**Figure 22:**
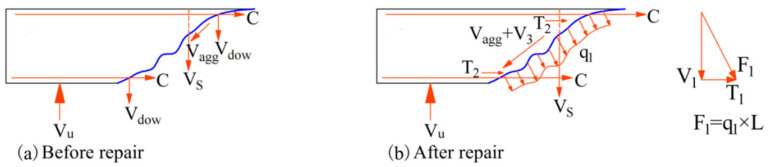
Isolated body force analysis.

To investigate the changes in the shear load-bearing mechanism before and after the repair of diagonal cracks using encapsulant adhesives, a stress analysis was conducted along the direction of the diagonal crack, as illustrated in Figure 22. At this location, the shear force is primarily transferred through three mechanisms: the shear force V_agg_ transmitted via aggregate interlock, the shear force V_s_ borne by the hoop reinforcement, and the dowel action V_dow_ provided by the longitudinal reinforcement to resist shear slip along the cracked surface. Among these, the contribution of V_dow_ to the overall shear transfer is relatively limited.

After grouting repair, a non-uniform vertical load (q1) is generated on the concrete interface perpendicular to the diagonal crack during the secondary loading process because of the high positive tensile adhesion strength of the potting adhesive. The length of the diagonal crack is L, and the resultant force is F_1_ = q_1_ × L. F_1_ can be broken down into T_1_ (horizontal tensile force) and V_1_ (vertical shear force). The biting force of the aggregate V_agg_ is the primary mechanism that transferred shear force on the steel–concrete interface; the chemical adhesive force V_3_ acted as a secondary mechanism. The primary shear force is shared by V_1_, V_agg_, and V_3_, with the hoop reinforcement supporting just a minor portion of the shear force.

To evaluate the proportion of shear force borne by the stirrups relative to that carried by the concrete before and after repair, the shear forces resisted by the stirrups in different beam specimens during both the initial and secondary loading phases were calculated. The corresponding calculation formulas are presented in Equations (5) and (6).

(5) $$V_{s1}=E_{s}\varepsilon_{s1}A_{s}$$

(6) $$V_{s2}=E_{s}\varepsilon_{s2}A_{s}$$

where V_s1_ is the shear force carried by the hoop reinforcement for the first loading, E_s_ is the modulus of elasticity for the hoop reinforcement, E_s_ = 2.1 × 10^5^ N/mm^2^, ε_s1_ is the strain increment of the end hoop reinforcement after the second loading, A_s_ is the cross-sectional area of the reinforcing bar, A_s_ = 56.54 mm^2^, V_s2_ is the shear force carried by the hoop reinforcement for the second loading, and ε_s2_ is the strain increment of the end hoop reinforcement after the second loading.

The relevant parameters and calculation results of beams B-2, B-3, B-4, and B-5 are shown in Table 5.

The shear force contributions of the concrete and stirrups in different beam specimens are summarized in Table 6. As shown, after repair, the proportion of shear force carried by the stirrups in beams B-2, B-4, and B-5 decreased by 91.8%, 90.6%, and 90.7%, respectively, compared to their pre-repair values. Conversely, the proportion of shear force carried by the concrete increased by 39.3%, 30.7%, and 30.4%, respectively. These results indicate that following grouting repair, the primary shear-resisting mechanism shifted from the stirrups to the concrete. This shift improved the energy dissipation capacity of the beams and contributed to enhanced overall load-bearing performance.

After the repair of beam B-3, the proportion of shear force carried by the concrete decreased by 8.2% compared to the pre-repair condition. This reduction is attributed to the high viscosity of the grout, which hindered its penetration into the interface between the stirrups and the surrounding concrete. Consequently, the stirrups remained the primary load-bearing component under high shear forces.

According to the Code for Design of Concrete Structures (GB 50010-2010 [36]), the shear resistance of web-reinforced beams subjected to concentrated loads can be calculated using Equation (7). However, due to the redistribution of shear force between the stirrups and concrete following grouting repair, Equation (7) can be modified to account for this change. The revised shear resistance formula for web-reinforced beams after grouting repair is presented in Equation (8).

(7) $$V_{u}=\frac{0.2}{\lambda +1.5}f_{c}{bh}_{0}+1.25\frac{f_{yv}A_{sv}}{s}h_{0}$$

(8) $$V_{u}=\beta \frac{0.2}{\lambda +1.5}f_{c}{bh}_{0}+1.25\delta \frac{f_{yv}A_{sv}}{s}h_{0}$$

where V_u_ is the shear resistance of the stiffened beam, λ is the shear span ratio, f_c_ is the compressive strength of concrete in the axial direction, b is the width of the beam section, h_0_ is the effective height of the beam section, f_yv_ is the yield strength of the tensioned tie wire, A_sv_ is the cross-sectional area of the shear-resisting tie wire, s is the spacing of the tie wires along the length of the member, β is the coefficient of increase in concrete shear resistance, and δ is the coefficient of reduction in the tie wire shear resistance.

Table 7 shows the values of β and δ for beams B-2, B-4, and B-5. It can be observed that the range of β is between 1.304 and 1.394, and the range of δ is between 0.082 and 0.094.

However, due to cost and time constraints, as well as the limited number of test specimens, this study can only propose modifications to the existing theoretical formulations based on the identified repair mechanisms. The applicable parameter ranges require further investigation, refinement, and experimental validation in future research.

## 4. Conclusions

This study demonstrates that the composite modification of epoxy resins can significantly enhance their mechanical properties (tensile strength and elongation at break), in-service characteristics (viscosity and bond strength), and crack-repair performance. A clear correlation is observed between the effectiveness of concrete crack repair and the performance characteristics of the potting adhesive. Based on the experimental findings, the following conclusions can be drawn:

(1) The tensile strength, elongation at break, bond strength, and viscosity of the modified epoxy resin were evaluated. Through a combination of orthogonal experimental design and gray relational analysis, the optimal formulation of the composite-modified epoxy potting adhesive was identified. The results indicate that the composite adhesive exhibits the best overall performance when the contents of nano-TiO_2_, CTBN, and D-669 are 1%, 15%, and 9%, respectively, outperforming epoxy resins modified with individual components.
(2) The composite-modified epoxy resin with the optimized formulation exhibited notable improvements in mechanical properties compared to single-component-modified epoxy resins. Specifically, tensile strength, elongation at break, and bond strength increased by 4.07–21.16 MPa, 13.28–20.40%, and 1.05–3.79 MPa, respectively, while viscosity was reduced by 48–872 mPa·s. Structures repaired using the composite-modified epoxy resin demonstrated higher cracking loads and exhibited reduced crack widths and beam displacements under both monotonic and cyclic loading conditions. These findings confirm that composite modification effectively integrates the advantages of multiple materials, resulting in superior overall performance.
(3) The modified epoxy resin potting adhesive primarily functions within the load range between the initial cracking load and the ultimate load. In contrast, the ultimate load-bearing capacity of the reinforced concrete beams is predominantly governed by their internal reinforcement configuration.
(4) The viscosity of the potting adhesive is critical to its crack penetration and repair effectiveness; its toughness determines the potential for secondary cracking, and its bond strength significantly influences the stiffness recovery of the repaired structure.
(5) Following repair with the modified epoxy resin potting adhesive, a bonding layer was formed as the adhesive permeated the interface between the steel reinforcement and the surrounding concrete along the direction of the crack. This bonding layer facilitates chemical adhesion at the reinforcement–concrete interface, thereby restoring the strength of the cracked concrete and enabling it to participate in shear force transfer alongside the hoop reinforcement. Based on the experimental results, revised formulas for calculating the shear capacity of web-reinforced beams repaired with grouted adhesive under concentrated loading have been developed.

## Figures and Tables

**Table 1:** Test scheme.

Number	TiO_2_ Content/%	CTBN Content/%	D-669 Content/%
Z1	0.5	15	6
Z2	0.5	20	12
Z3	0.5	25	9
Z4	1.0	15	12
Z5	1.0	20	9
Z6	1.0	25	6
Z7	1.5	15	9
Z8	1.5	20	6
Z9	1.5	25	12

**Table 2:** GRG for composite-modified epoxy resin.

Number	Tensile Strength/MPa	Elongation at Break/%	Bond Strength/MPa	Viscosity /mPa·s	GRG
Z1	57.4	17.76	5.63	724	0.54
Z2	44.9	14.34	4.81	708	0.42
Z3	31.13	21.99	5.16	784	0.44
Z4	52.41	16.50	5.57	314	0.62
Z5	58.53	28.52	6.42	726	0.81
Z6	60.29	15.42	4.83	1074	0.47
Z7	64.21	11.6	4.96	520	0.59
Z8	52.11	19.53	6.28	404	0.68
Z9	48.67	17.22	4.73	674	0.45

**Table 3:** GRG for one-component-modified epoxy resin.

Number	Nano-TiO_2_ Content/%	CTBN Content/%	D-669 Content/%	Tensile Strength/MPa	Elongation at Break/%	Bond Strength/MPa	Viscosity /mPa·s	GRG
A1	0.5	0	0	60.11	7.42	3.70	739	0.52
A2	1.0	0	0	62.60	8.12	4.21	681	0.88
A3	1.5	0	0	61.03	5.31	3.94	656	0.58
A4	2.0	0	0	57.40	4.94	3.81	643	0.51
A5	2.5	0	0	54.25	4.49	3.56	629	0.50
B1	0	10	0	51.97	7.98	4.91	1342	0.69
B2	0	15	0	49.10	11.01	5.14	1473	0.59
B3	0	20	0	48.09	15.24	5.37	1601	0.77
B4	0	25	0	46.56	13.62	4.82	1926	0.48
B5	0	30	0	45.97	8.88	4.63	2322	0.34
C1	0	0	3	41.41	6.56	2.72	642	0.67
C2	0	0	6	39.56	8.12	2.68	425	0.64
C3	0	0	9	37.37	11.63	2.63	378	0.73
C4	0	0	12	33.57	7.47	2.57	343	0.54
C5	0	0	14	29.64	5.88	2.49	326	0.50

**Table 4:** Repair scheme.

Number	Repair Materials
B-1	-
B-2, B-6	A2
B-3, B-7	B3
B-4, B-8	C3
B-5, B-9	Z5

**Table 5:** Relevant parameters for shear capacity calculation.

	B-2	B-3	B-4	B-5
ε_s1_/10^−6^	1203.45	1191.04	1015.98	995.68
ε_s2_/10^−6^	105.01	1734.82	117.19	115.87
V_s1_/kN	14.29	14.14	12.06	11.82
V_s2_/kN	1.43	20.59	1.39	1.37

**Table 6:** Percentage of shear force on each component.

No.	Before Repair					After Repair				
	V_s_/kN	V_c_/kN	V_u_/kN	V_s_/V_u_/%	V_c_/V_u_/%	V_s_/kN	V_c_/kN	V_u_/kN	V_s_/V_u_/%	V_c_/V_u_/%
B-2	14.29	33.40	47.69	29.96	70.04	1.43	56.78	58.21	2.46	97.54
B-3	14.14	33.64	47.78	29.59	70.41	20.59	37.59	58.18	35.39	64.61
B-4	12.06	35.62	47.68	25.29	74.71	1.39	56.72	58.11	2.39	97.61
B-5	11.82	35.20	47.02	25.14	74.86	1.37	56.91	58.28	2.35	97.65

V_c_ represents the shear force carried by the concrete and the gel.

**Table 7:** Correction factor.

	B-2	B-4	B-5
β	1.393	1.307	1.304
δ	0.082	0.094	0.093

## Data Availability

The original contributions presented in this study are included in the article. Further inquiries can be directed to the corresponding author.
